# *Helicobacter pylori* Infection in Various ABO Blood Groups of Kashmiri Population

**DOI:** 10.1155/DTE.4.65

**Published:** 1997

**Authors:** Gh. Jeelani Romshoo, Md. Youssuf Bhat, G. M. Malik, Ab. Rasheed Rather, B. A. Naikoo, Javaid A. Basu, Tajamul Hussain, Samia Rashid

**Affiliations:** 1 Department of Medicine (G.I.T. Division) S.M.H.S Hospital Government Medical College Srinagar Srinagar Kashmir India; 2 c/o Post Box No. 757 G.P.O. Srinagar Kashmir 190 001 India

## Abstract

*Aim*: This study was carried out to assess the prevalence of *Helicobacter pylori* infection in various ABO blood groups of people of Kashmir.

*Method*: The study comprised 80 individuals – 50 peptic ulcer patients (whose disease was diagnosed by endoscopy) and 30 asymptomatic volunteers. Every subject's blood group and Rhesus status was determined by standard serological tests. *Helicobacter pylori* infection was diagnosed by three different methods viz., one minute endoscopy room test (urease test), Gram staining and by histology. The detection of *Helicobacter pylori* by histological examination using Giemsa staining was taken as the ‘gold standard’ for the presence of *Helicobacter pylori* infection.

*Results*: Out of 80 individuals, 67 were males and 13 females aged between 18–65 years. The majority of peptic ulcer patients had blood group ‘O’ (*n* = 28.56%). The prevalence of *Helicobacter pylori* infection amongst peptic ulcer patients was 76%. There was no difference in *Helicobacter pylori* positivity in various blood groups.

*Conclusion*: Blood group ‘O’ though a risk factor for peptic ulcer (Duodenal ulcer) is not a risk factor for acquiring *Helicobacter pylori* infection.


*Diagnostic and Therapeutic Endoscopy*, Vol. 4, pp. 65–67
Reprints available directly from the publisher
Photocopying permitted by license only

© 1997 OPA (Overseas Publishers Association)
Amsterdam B.V. Published under license
in The Netherlands under the Harwood Academic
Publishers imprint, part of The Gordon and
Breach Publishing Group.
Printed in Singapore.

### *Helicobacter pylori* Infection in Various ABO Blood Groups of Kashmiri Population

GH. JEELANI ROMSHOO*, MD. YOUSSUF BHAT, G.M. MALIK, AB. RASHEED RATHER, B.A. NAIKOO, JAVAID A. BASU, TAJAMUL HUSSAIN and SAMIA RASHID

*Department of Medicine (G.I.T. Division), S.M.H.S Hospital, Srinagar, Affiliated to Government Medical College Srinagar, Kashmir, India*

*(Received 23 May 1997; In final form 23 June 1997)*

***Aim*: This study was carried out to assess the prevalence of *Helicobacter pylori* infection in various ABO blood groups of people of Kashmir.**

***Method*: The study comprised 80 individuals – 50 peptic ulcer patients (whose disease was diagnosed by endoscopy) and 30 asymptomatic volunteers. Every subject's blood group and Rhesus status was determined by standard serological tests. *Helicobacter pylori* infection was diagnosed by three different methods viz., one minute endoscopy room test (urease test), Gram staining and by histology. The detection of *Helicobacter pylori* by histological examination using Giemsa staining was taken as the 'gold standard' for the presence of *Helicobacter pylori* infection.**

***Results*: Out of 80 individuals, 67 were males and 13 females aged between 18–65 years. The majority of peptic ulcer patients had blood group 'O' ($n = 28.56\%$). The prevalence of *Helicobacter pylori* infection amongst peptic ulcer patients was 76%. There was no difference in *Helicobacter pylori* positivity in various blood groups.**

***Conclusion*: Blood group 'O' though a risk factor for peptic ulcer (Duodenal ulcer) is not a risk factor for acquiring *Helicobacter pylori* infection.**

*Keywords:* ABO blood group, *Helicobacter pylori*, Duodenal ulcer, Volunteers

### INTRODUCTION

Duodenal ulcer is a heterogenous genetic disorder in which blood group 'O' and ABO non-secretor status has been regarded as a risk factor [1]. *Helicobacter pylori* (*H. pylori*) infection has been strongly implicated in the pathogenesis of antral gastritis (Type B), duodenal ulcer and recently gastric cancer [2,3]. Since antral colonisation with *H. pylori* has been observed in 95–100% of duodenal ulcer patients, blood group 'O' was thought to be a risk factor for acquiring *H. pylori* infection as well.

This study was undertaken to find out if there existed any association between ABO blood group and *H. pylori* infection amongst the Kashmiri

*Corresponding author. Address for Correspondence: c/o Post Box No. 757, G.P.O., Srinagar, Kashmir 190 001, India.

population (highly endemic for peptic ulcer disease).

### METHODS

This study conducted in the gastroenterology department of SMHS Hospital, Srinagar, (Kashmir), comprised of 80 individuals (67 males, 13 females in the age group of 18–65 years). ABO blood grouping and Rhesus status of each individual was determined with standard serological tests. Multiple punch biopsies were taken from gastric antrum for identification of *Helicobacter pylori* (*H. pylori*). Three different tests were used for diagnosis of *H. pylori* viz., one minute endoscopy room test (urease test), Gram staining and histology. Detection of *H. pylori* by histology using Giemsa stain was taken as the 'gold standard' for presence of *H. pylori* infection [4–6]. Individuals with history of ingestion of antibiotics or bismuth within six weeks period were excluded from the study.

Statistical analysis was done using the $x^2$ test. Informed consent was obtained from all individuals. This study was approved by the Board of Studies and Principal/Dean of the Government Medical College, Srinagar.

### RESULTS

Out of 80 subjects 50 were having peptic ulcer disease (DU = 46, GU = 2 and combined DU and GU = 2) and 30 asymptomatic volunteers. 48 (60%) subjects including both peptic ulcer patients and asymptomatic volunteers were *H. pylori* positive. Out of these 48 *H. pylori* positive individuals 40 (83.33%) were Rhesus D positive compared to 28 (87.50%) of those *H. pylori* negative individuals. The age, sex, blood group distribution of these individuals is shown in Tables I and II. The distribution of *H. pylori* positivity amongst various blood groups is shown in Table III and IV.

$x^2$ test analysis did not find any significant association between the blood group distribution and presence or absence of *H. pylori* infection in this study.

TABLE I Age and sex characteristics of the participants ($n = 80$)

| Group | Number | Age | Sex | |
|---|---|---|---|---|
| | | | Male | Female |
| Peptic ulcer patients | 50 | 18–65 yrs | 42 | 08 |
| Healthy volunteers | 30 | 18–65 yrs | 25 | 05 |

TABLE II Distribution of blood group in peptic ulcer patients and healthy volunteers

| Group | Number | Blood group | | | |
|---|---|---|---|---|---|
| | | A | AB | B | O |
| Peptic ulcer patients | 50 | 10 | 1 | 11 | 28 |
| Healthy volunteers | 30 | 11 | 3 | 7 | 9 |

TABLE III *Helicobacter pylori* positivity by histology in various blood groups of peptic ulcer and healthy volunteers

| Group | Blood group | Number | H. pylori positivity (%) |
|---|---|---|---|
| Peptic ulcer patients (50) | O | 28 | 23 (82.14%) |
| | A | 10 | 08 (80.00%) |
| | B | 11 | 07 (63.64%) |
| | AB | 01 | 0 (—) |
| Healthy volunteers (30) | O | 09 | 3 (33.33%) |
| | A | 11 | 4 (36.36%) |
| | B | 07 | 2 (28.57%) |
| | AB | 03 | 1 (33.33%) |

TABLE IV Overall *H. pylori* positivity in various blood groups by histology

| *H. pylori* status | Blood group | | | | Total |
|---|---|---|---|---|---|
| | O | A | B | AB | |
| Positive | 26 | 12 | 09 | 01 | 48 |
| Negative | 11 | 09 | 09 | 03 | 32 |

### DISCUSSION

Although $Le^b$ and H antigens were proposed to be receptors for the attachment of *H. pylori* to gastric mucosa [6] but our study like many other studies did not find any association between blood group phenotypes and the prevalence of *H. pylori* infection or *H. pylori* related diseases. Despite the fact that the majority of peptic ulcer disease patients (56%) had blood group 'O'.

Many studies carried out previously also did not find any association between *H. pylori* infection and ABO blood groups, but these studies had used either the rapid urease test procedure [7] or serological methods [8–10] for diagnosis of *H. pylori* infection.

The major drawback with serological test methods is that they do not detect acute *H. pylori* infection. So this study was carried out using the histological test method for diagnosis of *H. pylori* infection. The blood group of the individual had no bearing on the results of this study.

In summary, though blood group 'O' is a risk factor for duodenal ulcer it is not a risk factor for acquiring *H. pylori* infection, and blood group 'O' and *H. pylori* infection are two independent risk factors in the pathogenesis of duodenal ulcer disease.

### *References*

[1] Rotter, J.I. and Rimoin, D.L. Peptic ulcer disease – A heterogenous group of disorders? *Gastroenterology* 1977; **73**: 604–607.

[2] Graham, D.Y. Helicobacter pylori – its epidemiology and its role in duodenal ulcer disease. *J. Gastroenterol. Hepatol.* 1991; **6**: 105–113.

[3] David, Y. Graham and Nae, F. Go Helicobacter pylori: Current status. *Gastroenterology* 1993; **105**: 279–282.

[4] Barthel, J.S. and Evertt, E.D. Diagnosis of campylobacter pylori infection. The "gold Standard" and the alternatives. *Rev. infect. Dis.* 1990; **12**(Suppl): 5107.

[5] Mohmmed, A.E., Al-Karaw, A., Al-Jumah, A. *et al.* Helicobacter pylori: Incidence and comparision of three diagnostic methods in 196 saudi patients with dyspepsia. *Hepatogastroenterol.* 1994; **41**: 48–50.

[6] Boren, T., Falk, P., Roth, K.A. *et al.* Attachment of Helicobacter pylori to human gastric epithelium mediated by blood group antigens. *Science* 1993; **262**: 1892–5.

[7] Levi, S., Davies, K.A.A., Play Ford, R.A. *et al.* Antral campylobacter colonization, ABO blood group and secretor status. *Gastroenterol. Clin. Biol.* 1989; **13**: 1095.

[8] Hook-Nikanne, J., Sistonen, P. and Kosunen, T.U. Effect of ABO blood group and secretor status on the frequency of Helicobacter pylori antibodies. *Scand. J. Gastroenterol.* 1990; **25**: 815–18.

[9] Loffeld, R.J.L.F. and Stobberingh, E. Helicobacter pylori and ABO blood groups. *J. Clin. Pathol.* 1991; **44**: 516–517.

[10] Umlauft, F., Keefe, E.B., Offner, F. *et al.* Helicobacter infection and blood group antigens: lack of clinical association. *Am. J. Gastroenterology* 1996; **91**(10): 2135–2138.

